# SPECTRUM – A MATLAB Toolbox for Proteoform Identification from Top-Down Proteomics Data

**DOI:** 10.1038/s41598-019-47724-1

**Published:** 2019-08-02

**Authors:** Abdul Rehman Basharat, Kanzal Iman, Muhammad Farhan Khalid, Zohra Anwar, Rashid Hussain, Humnah Gohar Kabir, Maria Tahreem, Anam Shahid, Maheen Humayun, Hira Azmat Hayat, Muhammad Mustafa, Muhammad Ali Shoaib, Zakir Ullah, Shamshad Zarina, Sameer Ahmed, Emad Uddin, Sadia Hamera, Fayyaz Ahmad, Safee Ullah Chaudhary

**Affiliations:** 1 Biomedical Informatics Research Laboratory, Department of Biology, Lahore University of Management Sciences, Lahore, Pakistan; 2 Department of Computer Science, Lahore University of Management Sciences, Lahore, Pakistan; 3 King Abdullah University of Science and Technology, Thuwal, Saudi Arabia; 4 National Center for Proteomics, University of Karachi, Karachi, Pakistan; 5 Department of Mechanical Engineering, National University of Sciences and Technology, Islamabad, Pakistan; 6 Institute of Life Sciences, University of Rostock, Rostock, Germany; 7 Department of Statistics, University of Gujrat, Gujrat, Pakistan; 8 Lahore University of Management Sciences, Lahore, Pakistan

**Keywords:** Computational platforms and environments, Proteome informatics

## Abstract

Top-Down Proteomics (TDP) is an emerging proteomics protocol that involves identification, characterization, and quantitation of intact proteins using high-resolution mass spectrometry. TDP has an edge over other proteomics protocols in that it allows for: (i) accurate measurement of intact protein mass, (ii) high sequence coverage, and (iii) enhanced identification of post-translational modifications (PTMs). However, the complexity of TDP spectra poses a significant impediment to protein search and PTM characterization. Furthermore, limited software support is currently available in the form of search algorithms and pipelines. To address this need, we propose ‘SPECTRUM’, an open-architecture and open-source toolbox for TDP data analysis. Its salient features include: (i) MS2-based intact protein mass tuning, (ii) *de novo* peptide sequence tag analysis, (iii) propensity-driven PTM characterization, (iv) blind PTM search, (v) spectral comparison, (vi) identification of truncated proteins, (vii) multifactorial coefficient-weighted scoring, and (viii) intuitive graphical user interfaces to access the aforementioned functionalities and visualization of results. We have validated SPECTRUM using published datasets and benchmarked it against salient TDP tools. SPECTRUM provides significantly enhanced protein identification rates (91% to 177%) over its contemporaries. SPECTRUM has been implemented in MATLAB, and is freely available along with its source code and documentation at https://github.com/BIRL/SPECTRUM/.

## Introduction

Mass spectrometry-based proteomics is a well-established technique for protein identification, characterization, and quantitation^1–3^. The conventional Bottom-Up Proteomics (BUP)^4^ protocol involves mass spectrometry (MS) analysis of peptides obtained from enzymatic digestion of whole proteins^4,5^. Several software tools such as SEQUEST^6^, Mascot^7^ and ExPASy tools^8^ (FindPept^9^ and EasyProt^10^) have been reported for BUP data analysis. However, BUP spectra and its analysis have limited power in: (i) identification of post-translational modifications (PTMs)^2^, (ii) sequence coverage^11,12^, and (iii) characterization of very small proteins^13^. Recent advancements in proteomics protocols and instrumentation have enabled precise mass measurements of large proteins by employing soft ionization techniques^14^ coupled with high-resolution mass analyzers^15^. This has led to the emergence of Top-Down Proteomics^16^ (TDP) protocol which is becoming increasingly popular for analyzing intact proteins^17,18^. TDP offers an enhanced sequence coverage^19^ as compared to BUP^4^ along with an improved identification of proteoforms (proteins and its variants)^20,21^. However, the complexity of high-resolution TDP spectral data poses a significant challenge for analysis tools. Current tools for TDP include ProSight PTM^12^, ProSight PTM 2.0^22^, MS-Align+^23^, pTop^24^, TopPIC^25^, and MSPathFinder^26^ amongst others. ProSight PTM, the first tool reported for TDP data analysis, employed shotgun annotation^27^ for protein identification and PTM localization. ProSight PTM 2.0 enhanced ProSight PTM by providing an improved database annotation along with a capability to search variable, fixed as well as terminal modifications. However, the tool’s protein identification search space was limited to organism-specific protein sequence variations. Also, the shotgun annotation led to a significant increase in the size of search database. In 2012, MS-Align+ addressed this issue by using spectral alignment methodology^28^ to elicit unknown PTMs and truncated proteins. The tool, however, had a command line interface (CLI) rendering it difficult to use. In 2016, TopPIC and pTop were reported. TopPIC provided an improved implementation of MS-Align+ and facilitated high-throughput novel proteoforms discovery by including primary structure alterations. However, the tool was limited in its capability to identify proteins with multiple variable modifications. pTop, on the other hand, employed *de novo* sequencing to shortlist proteins and search combinations of user-provided variable modifications. This approach was particularly effective for searching multiple PTMs but was unable to cater for unknown modifications and truncated proteins. Recently reported MSPathFinder, a high-throughput tool employing parametric dynamic programming for spectral alignment, uses sequence graphs for efficient filtering of combinatorial proteoforms. However, it also lacks support for searching unknown modifications and its CLI makes it difficult to use. Taken together, TDP data analysis tools continue to suffer from limitations in: (i) identification of truncated proteins, (ii) identification, characterization and localization of unknown and multiple PTMs, (iii) identification of truncated proteoforms having PTMs, and (iv) an intuitive visualization of results. Moreover, the lack of open-architecture software practice impedes the development and benchmarking of TDP algorithms to address these shortcomings.

**Figure 1:**
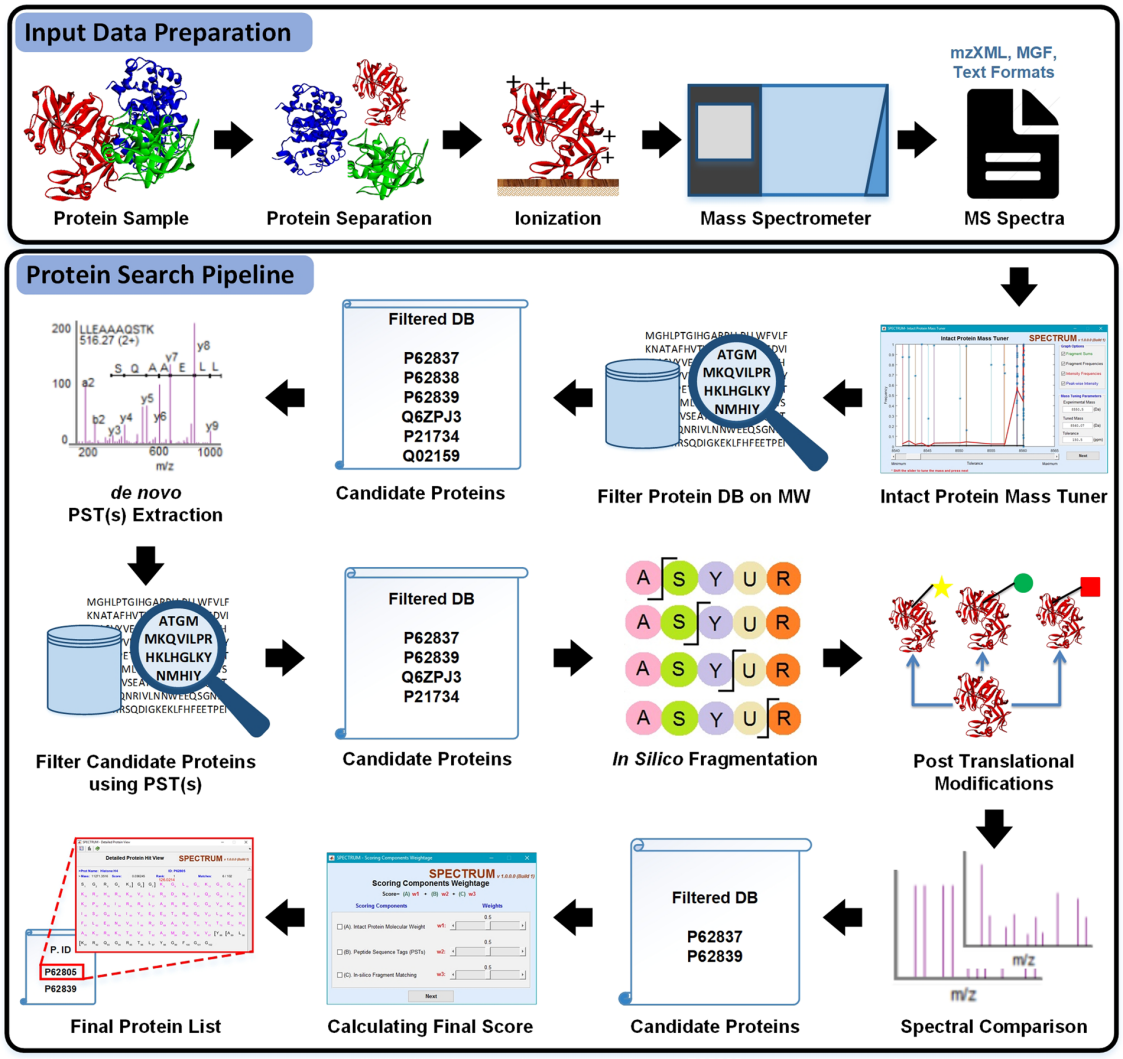
SPECTRUM workflow. The integrated experimental and computational data analysis pipeline employed in top-down proteomics.

In this work, we propose “SPECTRUM”, an open source and open architecture top-down proteoform identification toolbox for MATLAB. Several algorithms have been systematically integrated to form the core of SPECTRUM search pipeline (Fig. 1). These algorithms include a novel intact protein mass tuner to augment MS1 measurements for scoring and filtering protein databases. *De novo* sequencing has been employed for extracting and scoring peptide sequence tags (PSTs)^29,30^. A novel PTM prediction strategy employs dbPTM^31,32^ for evaluating the shortlisted candidate proteins for known PTM binding sites besides supporting a blind PTM search. SPECTRUM also provides search support for single-side truncated proteins. Lastly, the canonical spectral comparison between theoretical and experimental spectra^33–35^ has also been employed for refining candidate protein list. To develop an overall ranking of candidate proteins, a composite scoring scheme has been implemented wherein users can tune weights for individual component scores to obtain the final score. For data interoperability^36^, SPECTRUM currently supports plain text files (columns of mass to charge ratios (m/z) and relative intensities), eXtensible Markup Language (XML) files with m/z and relative abundances (mzXML)^37^, Mass Spectrometry Markup Language (mzML)^38,39^ and Mascot Generic Format (MGF)^7^ data formats in both single and batch file processing modes. Users can access the toolbox by a set of intuitive graphical user interfaces (GUIs) for setting up search parameters as well as viewing results. Each GUI has been developed using MATLAB GUI development environment (GUIDE)^40^ and can, therefore, be readily customized or refactored.

We have validated and benchmarked SPECTRUM toolbox by undertaking case studies on two published datasets. Case study I was performed to evaluate protein identification accuracy and blind PTM characterization using an experimental dataset^41^ with known target protein (HeLa Histone H4). Results obtained from SPECTRUM were compared with those from ProSightPC^42^ (a commercial version of ProSight PTM 2.0), TopPIC, and pTop. SPECTRUM correctly identified the target protein which was reported by ProSightPC and TopPIC (see Case Study I – Results Section). For evaluating SPECTRUM’s ability to identify unknown proteins, a second case study was carried out using an *Escherichia coli* dataset^25^. SPECTRUM results reported up to 47% more spectral matches and over 91% more proteins in comparison with other tools (see Case Study II – Results Section).

In conclusion, SPECTRUM is a state-of-the-art tool for protein identification and characterization and is available in the form of a conveniently customizable MATLAB toolbox. This open-architecture toolbox stands to impart impetus to the advancement of TDP by assisting in design, implementation and benchmarking of novel TDP algorithms leading to an improved proteoform identification.

## Results

In this work, we have reported SPECTRUM, a next-generation open-source MATLAB^40^ toolbox for top-down proteomics. The toolbox is available as a GitHub repository. Documentation (see Supplementary Information –E. Availability) and video tutorials have also been made available (see Supplementary Information –F. Video Tutorials).

**Figure 2:**
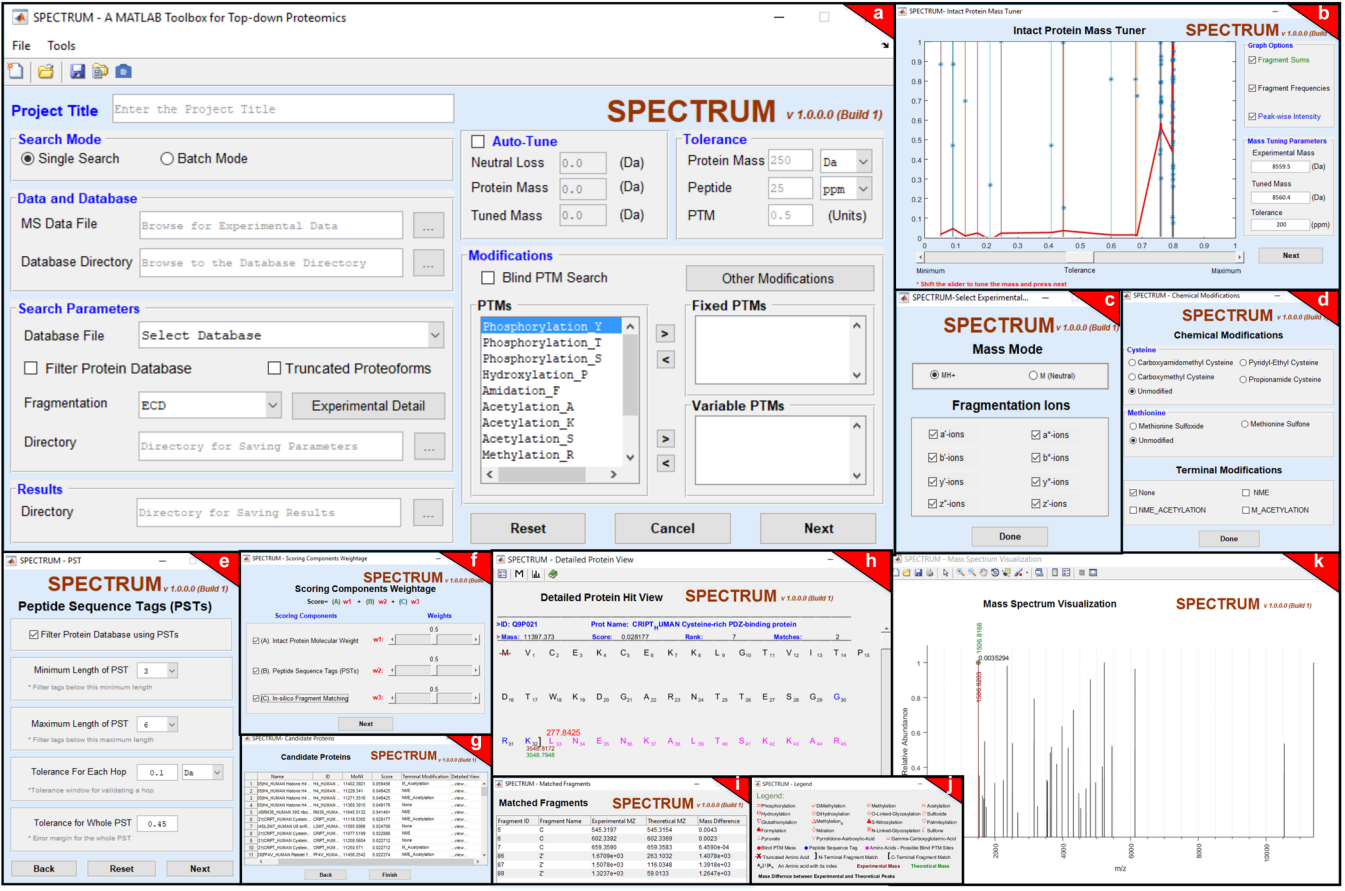
Overview of SPECTRUM GUIs. The set of graphical user interfaces (GUIs) in SPECTRUM created using MATLAB GUIDE to undertake the search process and visualize results. (**a**) Main SPECTRUM GUI to provide general search parameters, (**b**) GUI to tune intact protein mass, (**c**) GUI to specify special fragmentation ions and mass mode in the search process, (**d**) GUI to provide peptide sequence tag (PST) search parameters, and (**e**) GUI to specify instrument-based chemical modification(s) along with terminal modifications. (**f**) GUI to adjust weights in the scoring scheme, (**g**–**h**) GUIs to provide users with brief as well as detailed results, (**i**) GUI to describe spectral matching details, (**j**) GUI providing a legend for use in detailed result view, and (**k**) GUI for mass spectrum visualization.

The toolbox provides a comprehensive graphical user interface (GUI) framework (Fig. 2). The main GUI window (Fig. 2a) acts as the entry-point for setting up spectral data, protein databases, and search parameters. Elaborate GUIs have been provided for each step in the search process (Fig. 2b–f) and the summary of search results can be visualized as a ranked protein list (Fig. 2g). Using the “Detailed Protein View” (Fig. 2h), users can also view details of candidate proteins including information on predicted modifications, peptide sequence tags (PSTs) and theoretical fragments (Fig. 2i,k).

### Salient search features and algorithms of SPECTRUM

SPECTRUM’s top-down protein search pipeline comprises of three major components, i.e. (a) intact protein mass tuner and filter, (b) *de novo* sequencing and PST filter, and (c) *in silico* spectral comparator. SPECTRUM provides search support for chemical, terminal, fixed and variable modifications along with terminally truncated proteoforms. A blind post-translational modification (PTM) search module has also been included to search for unknown PTMs without requiring prior information. Data file format support for MGF^7^, mzXML^36,43^, mzML^38,39^ and flat text peak list file has been provided (see Supplementary Information – H. Feature Comparison). Alongside, SPECTRUM supports search in single as well as batch modes. Single-mode permits the users to search the four file formats while batch-mode allows for an automated search of multiple flat text files. Lastly, a multifactorial and customizable scoring scheme has been designed to tune the search process by weighing each component of protein search pipeline towards calculating the final scores.

### Case Study I – Evaluation of SPECTRUM search with known target protein

To validate the protein identification accuracy of SPECTRUM, we searched a HeLa spectral dataset^41^ with known target protein (Histone H4). The dataset consisted of ten files containing monoisotopic data (see Supplementary Data S1). The search results obtained from SPECTRUM were compared with pTop^24^, TopPIC^25^ and ProSightPC^22,42^ (see Supplementary Data S2). Target protein’s rank in the candidate protein list and search runtime were then obtained and compared. The first comparison was performed between SPECTRUM and pTop wherein *de novo* sequencing was employed (search parameters in Supplementary Table S1). pTop took 13 seconds to perform protein search, however, it failed to identify any protein from the dataset. SPECTRUM on the other hand, completed the search in 28 seconds and reported Histone H4 as the top-ranked protein in eight out of ten experiments. SPECTRUM did not report any protein for remaining two files (summary and complete results in Supplementary Tables S2 and S3, respectively). Next, we compared SPECTRUM with TopPIC, a spectral alignment tool (search parameters in Supplementary Table S4). TopPIC took 2350 seconds and reported Histone H4 for seven data files; one file reported a false positive and two did not report any protein. SPECTRUM took 21 seconds to search the complete dataset and correctly identified the true protein from eight data files while false positives were reported for the remaining two files (summary and complete results in Supplementary Tables S5 and S6, respectively). We then compared spectral comparison capability of SPECTRUM with ProSightPC (search parameters in Supplementary Table S7). For this purpose, PST-based filtering was disabled, and the weight of intact protein mass score was set to zero. ProSightPC completed the search in 24 seconds and reported Histone H4 as top-ranked protein for eight data files while false-positives were reported for the remaining two. SPECTRUM executed the search in 19 seconds and reported eight true-positives besides two false-positive entries (summary and complete results in Supplementary Table S8 and S9, respectively). An overall comparison of the search results obtained from each tool has been provided in Supplementary Table S10.

Having validated protein identification, we then evaluated SPECTRUM’s blind PTM search feature for identifying unknown PTMs without prior information from the user. TopPIC reported unknown mass shifts for seven correct identifications but could not translate them into PTMs. SPECTRUM not only captured these mass shifts but also successfully characterized PTMs from three data files (see Supplementary Table S11 and Supplementary Information – B. Supplementary Results).

To evaluate the sensitivity of the search process to various parameters, a sensitivity analysis was performed on intact mass, PST and *in silico* comparison components. The parameter variations used for intact protein mass tolerance were 250, 500, 1000 and 2000 Da, PST lengths between 4 to 6 and 3 to 6, and *in silico* spectral comparison tolerances of 15 and 25 ppm, respectively. By increasing PST length range, an improvement in protein identification was observed. However, variations in protein mass tolerance had a minimal impact. The results from parameter sensitivity have been tabulated in Supplementary Table S12 (also see Supplementary Information – B. Supplementary Results: Case Study I).

### Case Study II – Evaluation of SPECTRUM search with unknown target protein

After validating SPECTRUM search accuracy with known target proteins, we employed the toolbox to search a dataset with unknown target protein(s). Published *Escherichia coli* dataset^25^ obtained using alternating CID and ETD fragmentation modes (see Supplementary Data S3) was employed for the search. The search parameters have been provided in Supplementary Tables S13 and S14 for search with and without PSTs, respectively. The results were compared with those from MSPathFinder^26^, TopPIC^25^, and pTop^24^ at 1% false discovery rate and E-value of 1E-10 (summary of overall results in Supplementary Table S15).

The first comparison in this case study was performed between SPECTRUM and MSPathFinder. Peptide sequence tag (PST) filter was enabled for both the tools. SPECTRUM identified 245 proteins as compared to MSPathFinder which identified 128 proteins, indicating a 91% improvement. SPECTRUM also demonstrated an enhancement in number of PrSMs (1739) in comparison with MSPathFinder (1458). Next, the PST filter was turned off and the search was performed again. SPECTRUM reported 305 proteins and 1911 PrSMs in comparison to MSPathFinder’s 110 proteins and 1319 PrSMs, an improvement of 177% and 44% in proteins and PrSMs, respectively. We then compared SPECTRUM with TopPIC. Since TopPIC does not support tag-based search, SPECTRUM’s PST filter was disabled. SPECTRUM identified 305 proteins as compared to TopPIC which identified 128 proteins, indicating a 138% improvement. In comparison with 1911 PrSMs reported by SPECTRUM, TopPIC reported 1262 PrSMs. Lastly, we compared SPECTRUM toolbox with pTop. Since pTop’s search employs PSTs, we enabled SPECTRUM’s PST filter to search the dataset. SPECTRUM reported 245 proteins while pTop reported 128 proteins, marking a 91% improvement. Moreover, SPECTRUM reported 1739 PrSMs as compared to 1181 PrSMs from pTop, a 47% improvement.

**Figure 3:**
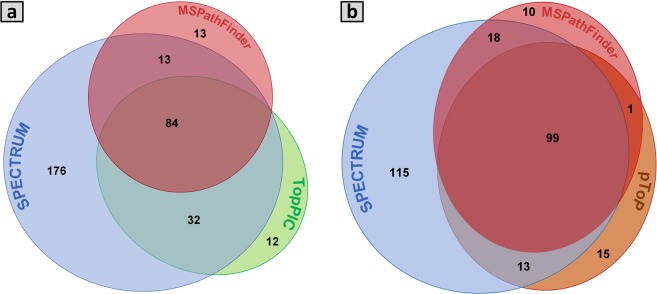
Venn diagrams exhibiting protein identification count in case study II. (**a**) The number of identified proteins by SPECTRUM, TopPIC and MSPathFinder without using PST filter. (**b**) The number of identified proteins by SPECTRUM, pTop and MSPathFinder after applying PST filter.

Taken together, SPECTRUM identified a significantly larger number of proteins as compared to MSPathFinder, TopPIC, and pTop from *Escherichia coli* dataset (Fig. 3). A summary of search results has been provided in Fig. 3 and Supplementary Table S15. The complete results for both target and decoy databases search for each fragmentation mode (CID and ETD) have been provided in Supplementary Tables S16–S23. A summary table listing the result files has been provided in Supplementary Information – B. Supplementary Results: Case Study II.

## Discussion

High-resolution top-down proteomics (TDP) is increasingly being employed for understanding mechanisms underpinning disease towards biomarker discovery^21,44–46^. Specifically, information-rich top-down mass spectra have a significant potential towards an enhanced proteoform identification^47^. For an optimal searching of TDP data, continuous advancement in top-down search algorithms and software is required. Contemporary tools for TDP have achieved remarkable protein identification rates, however, these tools provide partial search pipelines, are closed source or only available commercially. Besides, there is still a significant room for improvement in protein identification and characterization.

Towards addressing this need, we have proposed SPECTRUM, an open-source and open-architecture MATLAB toolbox for proteoform identification in top-down proteomics. SPECTRUM algorithmic pipeline advances the state-of-the-art by significantly enhancing proteoform identification and characterization as compared to the contemporary TDP tools (see Supplementary Table S24). To demonstrate the search capabilities of SPECTRUM, two case studies were conducted using published data^25,41^. In the first study, SPECTRUM successfully identified the known target protein, Hela - Histone H4, as was reported by pTop, ProSightPC and TopPIC. In the second study on *Escherichia coli* dataset with unknown target proteins, SPECTRUM reported up to 177% more proteins over other tools. Computational runtimes for the toolbox were also profiled and compared with MSPathFinder, pTop and TopPIC, for each case study. SPECTRUM runtimes were comparable with other tools for the HeLa dataset which comprised of 10 files^41^. However, for the larger *Escherichia coli* dataset, SPECTRUM runtime lagged behind other tools which can be attributed to the MATLAB interpreter. This can, however, be overcome by parallelizing the toolbox or by using MATLAB GPU computing routines. The blind PTM search module of SPECTRUM also improves upon TopPIC^25^ (see Supplementary Information – B. Supplementary Results) with an enhanced mass-shift identification and characterization. In terms of parameter sensitivity, three core modules including intact mass filter, peptide sequence tags (PST) generator and *in silico* spectral comparator influence the search to varying degrees (see Supplementary Information – B. Supplementary Results). Specifically, results were improved by increasing the range of PST length while no significant effect was observed for intact protein mass and spectral comparison. Prospectively, SPECTRUM can provide a significantly enhanced proteoform identification to its users. The batch-mode search also adds a high-throughput capability. Fixed, variable and blind modifications can be characterized besides reporting unexplained mass shifts. SPECTRUM pipeline also caters for truncated protein search. Users can customize the scoring scheme towards sensitizing the search process to their experimental setups. The graphical user interface (GUI) can be conveniently modified or enhanced using MATLAB GUIDE.

As with other spectral analysis tools, search results from SPECTRUM are dependent on the quality of MS data. Hence, the accuracy of search results may vary with mass spectrometer resolution. In terms of limitations, since SPECTRUM has been implemented in MATLAB, it requires a MATLAB license, thereby impeding the non-MATLAB users to run SPECTRUM. This need has been met with provision of the toolbox in form of an executable file (see Supplementary Information – E. Availability). SPECTRUM currently offers one-sided truncation and does not accommodate for double-sided truncations and amino acid substitutions. SPECTRUM’s blind-PTM module only characterizes those PTMs which are supported by spectral data. A natural extension will be incorporation of a probabilistic model in blind-PTM module for enhanced PTM characterization. Proteoform identification can be further enhanced by using combined spectral data obtained from alternating fragmentation mode of mass spectrometers. A useful extension of the toolbox can also come in the form of relative and absolute protein quantitation.

In conclusion, SPECTRUM is a state-of-the-art MATLAB-based top-down proteomics (TDP) toolbox that has been developed with an aim to assist in next-generation mass spectrometry data analysis. The toolbox is capable of identifying a significantly larger number of proteins as compared to its contemporaries besides characterizing post-translational modifications without requiring any prior knowledge. The proposed toolbox has been developed to facilitate biomedical research along with assisting in proteomics education by providing a versatile training platform for proteoform identification.

## Material and Methods

### Methodology and flow of SPECTRUM search pipeline

**Figure 4:**
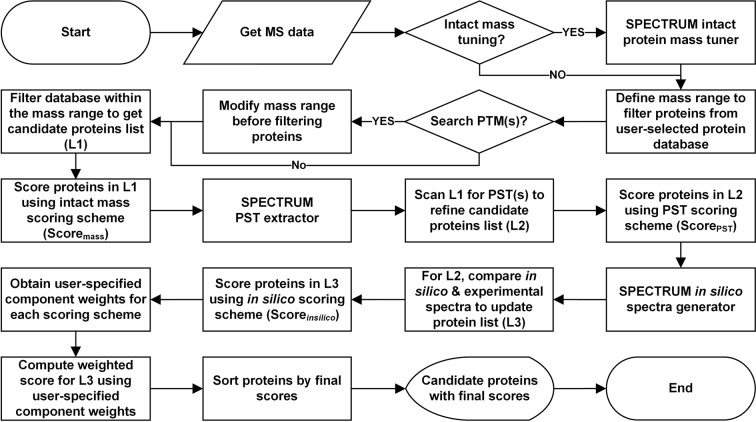
SPECTRUM data processing flowchart. User-selected protein database is filtered on intact protein mass followed by scoring of shortlisted proteins. *De novo* sequencing is performed to obtain peptide sequence tags (PSTs). Each candidate protein from the database is evaluated and scored for these sequence tags. Experimental and theoretical spectra of each candidate protein are compared to obtain *in silico* component score. Intact protein mass, PST and *in silico* scores are then used to determine final protein rank.

MATLAB 2017a^40^, a popular scientific computing platform, was used to develop SPECTRUM. A set of interactive GUIs were constructed using MATLAB graphical user interface (GUI) development environment (GUIDE)^40^ for taking user parameters and displaying search results. Figure 4 represents the overall methodology employed by SPECTRUM to search TDP data. Details on SPECTRUM search methodology, scoring scheme, validation, and data conversion have been provided below.

### SPECTRUM search methodology and scoring algorithms

#### Intact protein mass tuner

**Figure 5:**
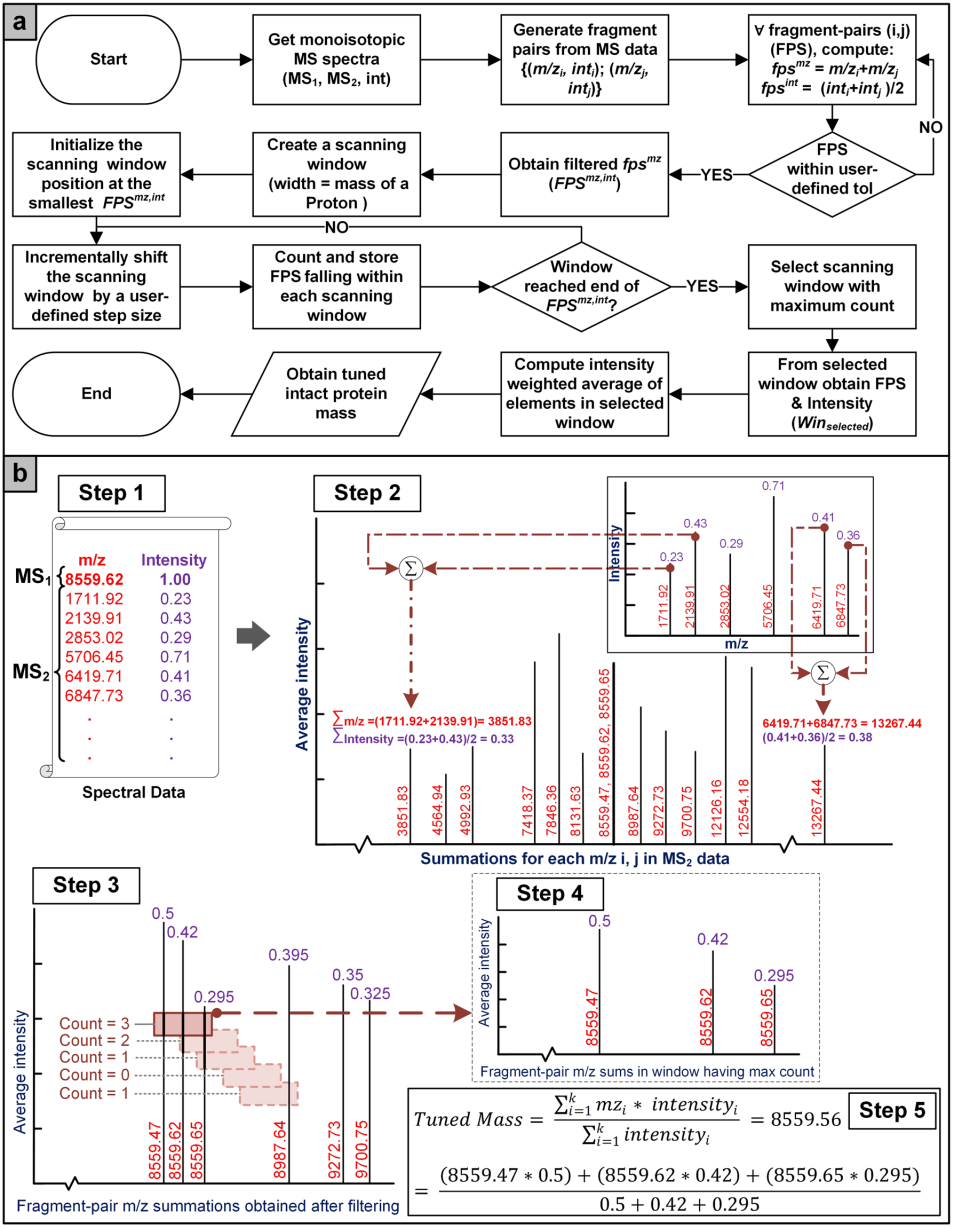
Intact protein mass tuner workflow. (**a**) Fragment-pair sums of MS2 data are computed and sorted in an ascending order. Sliding window of a size equal to the mass of a proton is used to determine the window with maximum number of peaks. Finally, tuned mass is obtained by calculating the intensity weighted average of tuple sums from selected window. (**b**) Contextual explanation of intact protein mass tuner, Step 1: Obtain experimental spectrum, Step 2: Compute fragment-pair sums of MS2 data, Step 3: Sliding window of size equal to mass of a proton is used to determine the number of peaks in each window, Step 4: Obtain window with maximum peak count, and Step 5: Tuned mass is obtained by calculating the intensity weighted average of tuple sums from selected window.

MS2 data comprising of mass to charge ratios of intact protein’s fragments and relative abundances, was used to tune the intact protein mass, MS1. Fragment-pairs were generated for each element in MS2 data and a tuned precursor whole protein mass (MS1) was computed from a sum of each pair. The fragment-pair sums within the user-defined tolerance were selected (*FPS*^*mz*^). The average of abundances for each shortlisted constituent element in *FPS*^*mz*^ were also computed. A window of size equal to the mass of a proton was used to scan the sorted fragment-pair sums to obtain the tuned mass. The window was progressively shifted by a user-defined step size and the number of fragment-pair sums falling within each window, at each shift, were counted. The window with the highest number of fragment-pair sums was selected, and tuned mass was computed as the intensity weighted average of fragment-pair sums within this window. A conceptual outline of the methodology has been shown in Fig. 5 and the complete set of mathematical equations have been provided in Supplementary Methods A1 - Intact Protein Mass Tuner.

#### Scoring proteins by intact protein mass

The absolute differences between theoretical masses (details in Supplementary Methods A2 - Computing Theoretical Mass of a Protein) of candidate proteins and the experimental mass (tuned mass or MS1) were calculated towards computing the protein score using intact protein mass. The proteins with mass difference within the user-defined tolerance were shortlisted and scored (equations (1, 2)).

1 $$Mas{s}_{diff}=|Mas{s}_{experimental}-Mas{s}_{theoretical}|$$

where,

*Mass*_*diff*_ is absolute difference between theoretically calculated mass of protein and experimental mass, *Mass*_*experimental*_ is experimental mass of sample protein (tuned mass or MS1), and *Mass*_*theoretical*_ is theoretical protein mass calculated using protein sequence.

2 $$Scor{e}_{mass}=\{\begin{array}{cc}1 & if\,Mas{s}_{diff}=0\\ {2}^{\frac{1}{Mas{s}_{diff}}} & \,if\,0 < Mas{s}_{diff}\le Thr\\ 0 & if\,Mas{s}_{diff} > Thr\end{array}$$

where,

*Score*_*mass*_ is the mass score of shortlisted protein, and *Thr* is user-defined intact protein mass tolerance.

#### Methodology for extracting peptide sequence tags

**Figure 6:**
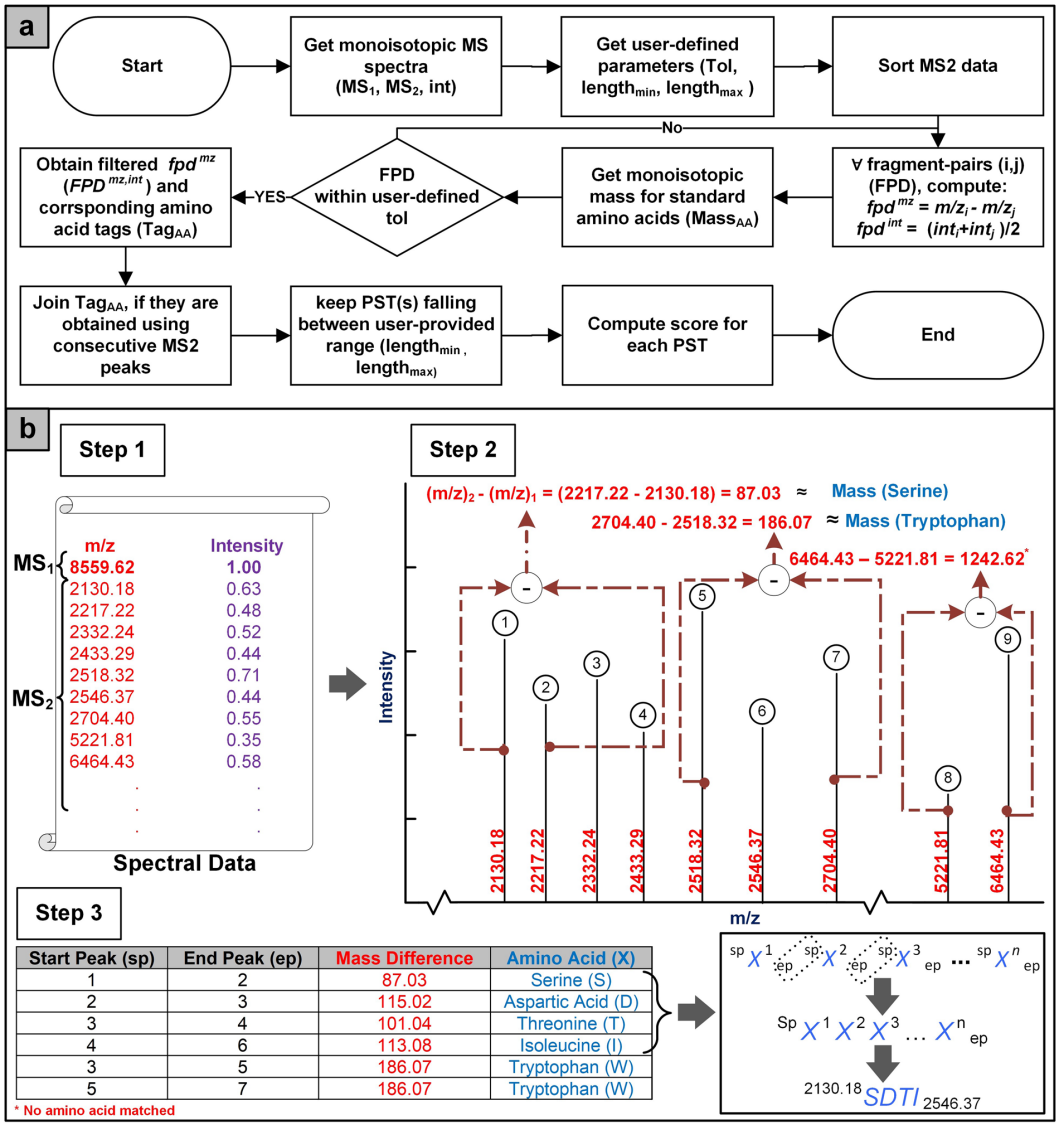
Workflow of peptide sequence tags (PSTs) extraction. (**a**) *De novo* sequencing of experimental data is performed to obtain peptide sequence tags. Each candidate protein from database is evaluated and scored for these PSTs. (**b**) Contextual explanation, Step 1: Obtain experimental spectrum, Step 2: Compute fragment-pair difference of MS2 data, Step 3: Obtain amino acids corresponding to fragment-pair differences, and Step 4: Tags having the same starting and ending peaks are joined together.

*De novo* sequencing was used to construct peptide sequence tag (PST) ladders. Incorporation of PSTs in the database search provided for tandem scoring of the candidate proteins. PST extractor was designed to take mass differences between successive experimental peaks within a user-specified tolerance. The mass difference corresponding to mass of any of the twenty amino acid residues constituted an amino acid tag. User-provided tolerance was used to determine the matching stringency for hops that mismatch the monoisotopic molecular weights of amino acids. The hops, with the starting peaks, ending peaks, the mass difference between these peaks, matching amino acid names and their molecular weights were stored. Hops having equal starting peak and ending peak values were joined together to form PST ladders. User-provided range of PST lengths was used to filter out anomalous (i.e. very short or very long) PST ladders to avoid biasing of the protein search process. The methodology is outlined in Fig. 6 and complete details have been provided in Supplementary Methods A3 - Extraction of Peptide Sequence Tags.

#### Scoring proteins using peptide sequence tags

PST scoring utilizes cumulative root mean squared error, peak intensities, PST occurrence count and PST length. *RMSE* over the entire PST length was computed and employed for shortlisting PSTs by user-defined tolerance. For each filtered tag, intensity of the constituent amino acids was determined by taking the average intensity of representative experimental peaks. Cumulative intensity of tag was then computed using average intensities for scoring. The influence of PSTs towards protein filtering and scoring was implemented to increase exponentially with length. The PST-based score for shortlisted proteins was computed using the frequency score, accumulative tag error score and occurrence of PST tags that reported these proteins. The scoring process has been defined in equations (3–10).

3 $$Erro{r}^{AA}=(Mas{s}_{experimental}-Mas{s}_{monoisotopic})$$

where,

*Error*^*AA*^ is the difference between *Mass*_*experimental*_ and *Mass*_*monoisotopic*_, *Mass*_*experimental*_ is the experimental mass of a residue present in an extracted PST, and *Mass*_*monoisotopic*_ is the monoisotopic mass of a standard amino acid residue in the PST.

4 $$RMSE=\frac{\sqrt{{\sum }_{i=1}^{N}\,{(Erro{r}_{i}^{AA})}^{2}}}{N}$$

where,

*RMSE* is cumulative root mean squared error calculated over the entire PST length, $Erro{r}_{i}^{AA}$ is the difference between experimental and theoretical mass of *i*^th^ residue in the PST, and *N* is length of peptide sequence tag.

5 $$Erro{r}_{score}=1/{e}^{2RMSE}$$

where,

*Error*_*score*_ is the cumulative score of PST error computed using *RMSE*.

6 $$in{t}_{PST}=(\frac{in{t}_{hop}+in{t}_{home}}{2})$$

where,

*int*_*PST*_ is the average intensity of constituent amino acids of PST; *int*_*home*_ and *int*_*hop*_ are the intensities of the peaks in the PST ladder.

7 $$Intensit{y}_{PST}=\frac{{\sum }_{i=1}^{N}\,in{t}_{PST}}{N}$$

where,

*Intensity*_*PST*_ is the cumulative intensity of all the amino acids in the PST.

8 $$Le{n}_{score}={N}^{2}$$

where,

*Len*_*score*_ is the score for length of a tag.

9 $$Fre{q}_{score}=Intensit{y}_{PST}\times Le{n}_{score}$$

where,

*Freq*_*score*_ is the PST component score computed using *Intensity*_*PST*_ and *Len*_*score*_.

10 $$Scor{e}_{PST}=\sum _{i=1}^{M}\,Occurenc{e}_{i}\times (Erro{r}_{Scor{e}_{i}}+Fre{q}_{Scor{e}_{i}})$$

where,

*Score*_*PST*_ is the PST score of shortlisted proteins, *Occurence* is the frequency of occurrence of a PST tag in a protein sequence, and *M* is the total number of tags.

#### Spectral generation and comparisons

A total of nine fragmentation techniques including collision-induced dissociation (CID), electron-capture dissociation (ECD), electron-transfer dissociation (ETD) and electron-detachment dissociation (EDD) etc. have been employed in SPECTRUM search pipeline. Additionally, single-sided truncations have also been incorporated. The mass of N-terminus ion was computed by summing up the masses of its constituent amino acids while for C-terminus ion, the mass was obtained by calculating the mass difference between the N-terminus ion and protein molecular weight. Also, during fragmentation, a hydroxyl group and a proton were added to N-terminus ion and C-terminus ion, respectively (see Supplementary Methods A4 - Spectral Generation and Comparison). User-specified neutral ion loss parameters were used to cater for fragments which have gained or lost functional groups.

**Figure 7:**
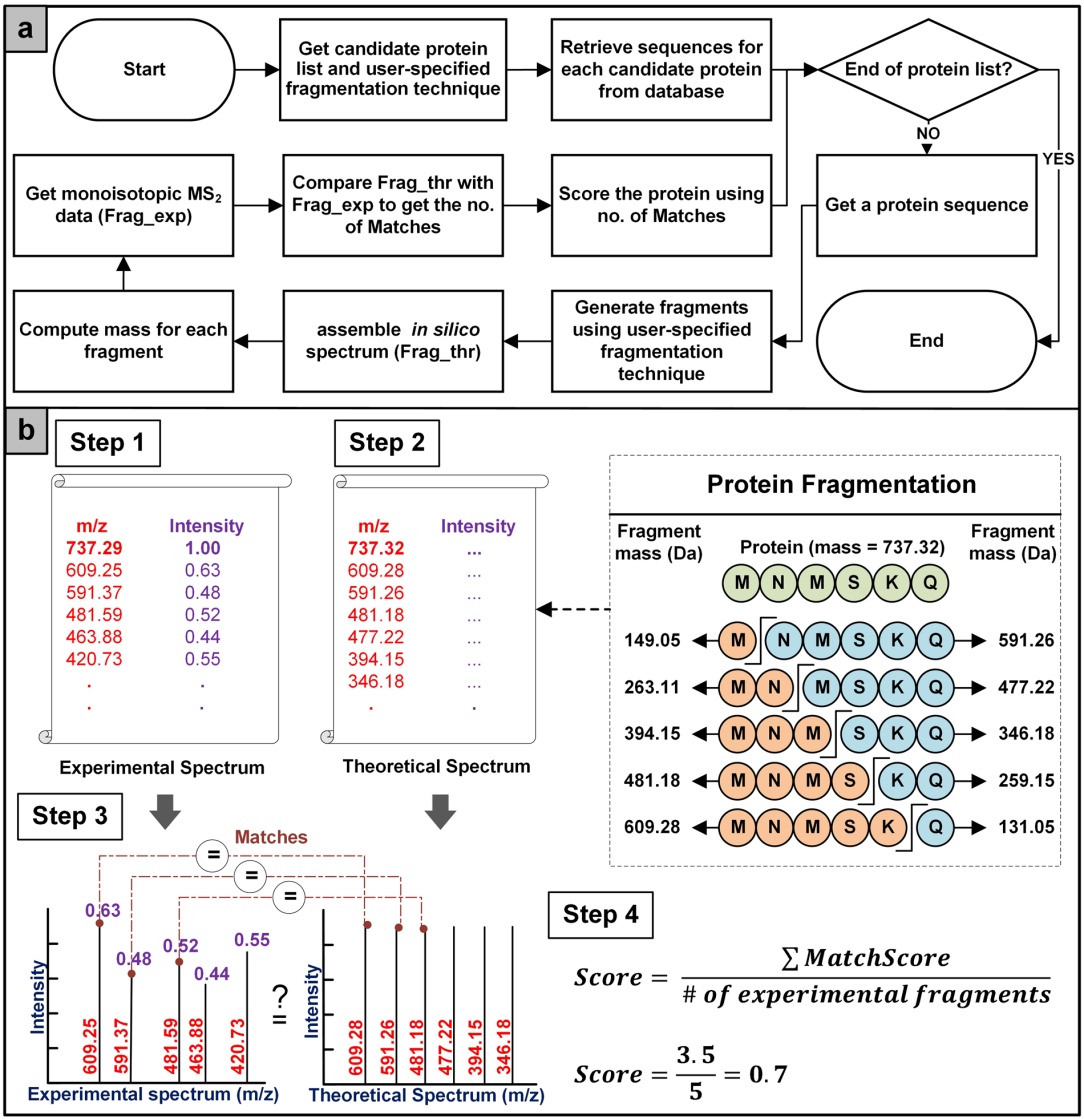
Spectral generation and comparisons workflow and contextual explanation. (**a**) After retrieving protein sequences from user-selected protein database, theoretical fragments of each protein are generated. Experimental and theoretical spectra are then compared to get *in silico* component score. (**b**) Step 1: Obtain experimental spectrum, Step 2: Generate theoretical fragments of candidate protein, Step 3: Experimental and theoretical spectra are compared to get number of matches, and Step 4: *In silico* component score is computed.

For a given experimental dataset, its intensity values were normalized between 0 and 1 followed by their scaling (*NormalizedIntensity*) using a step function described in equation (11). Note that the threshold of 9.2 × 10^−5^ was set after performing a sensitivity analysis on several available spectral datasets. Towards scoring the proteins using the *in silico* spectrum, N-terminus ions and C-terminus ions were compared with the experimental data within a certain user-specified tolerance. For every match, the candidate protein was awarded a score, based on the number of consecutive fragment matches in experimental spectrum (*ConsecutivePeakCounter*), as shown in equation (12). Next, the final score was computed for each protein using equation (13). The process has been outlined in Fig. 7.

11 $$NormalizedIntensity=\,\{\begin{array}{ll}0.001 & if\,Intensity < 9.2\times {10}^{-5}\\ 1 & if\,Intensity\ge 9.2\times {10}^{-5}\end{array}$$

where,

*NormalizedIntensity* is the scaled intensity value of experimental spectrum, and *Intensity* is the intensity of experimental spectrum normalized to 1.

12 $$MatchScor{e}_{i}=\{\begin{array}{ll}NormlizedIntensit{y}_{i} & if\,ConsecutivePeakCounter < 3\\ 1.5\, & \,if\,ConsecutivePeakCounter\ge 3\end{array}$$

where,

*MatchScore*_*i*_ is the score of fragment match corresponding to *i*^th^ experimental peak, *NormlizedIntensity*_*i*_ is the sigmoid weighted intensity value of *i*^th^ experimental peak, and *ConsecutivePeakCounter* is the number of consecutive experimental peak matches.

13 $$Scor{e}_{insilico}=\frac{{\sum }_{i=1}^{n}\,MatchScor{e}_{i}}{Fra{g}_{experimental}}$$

where,

*MatchScore*_*i*_is the score of *i*^th^ fragment match, *Frag*_*experimental*_ is the total number of experimental fragments, and *n* is the number of spectral matches.

#### Composite scoring scheme

The candidate protein list was ranked using (i) intact protein mass filtering (*Score*_*mass*_), (ii) PST filtering (*Score*_*pst*_) and (iii) spectral matching (*Score*_*insilico*_). The weight of each scoring component can be adjusted towards sensitizing the scoring to their experimental settings using equation (14).

14 $$Scor{e}_{final}=\frac{(Scor{e}_{mass}\times {W}_{1})+(Scor{e}_{PST}\times {W}_{2})+(Scor{e}_{insilico}\times {W}_{3})}{3}$$

where,

*Score*_*final*_ is the final score for each candidate protein shortlisted from the database, *W*_1_ is the weight set by the user for intact protein mass score, *W*_2_ is the weight set by the user for PSTs score, and *W*_3_ is the weight set by the user for *in silico* score. Note that the default weight (‘1’) elicits maximal sensitivity from each scoring sub-system in SPECTRUM.

#### Methodology for predicting post-translational modifications

**Figure 8:**
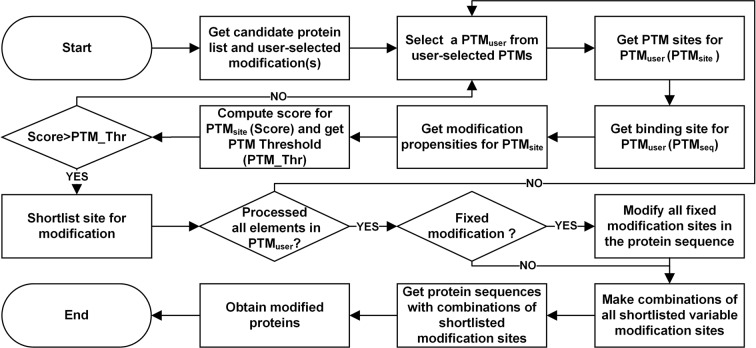
Prediction of post-translational modifications. SPECTRUM predicts fixed and variable post-translational modifications. The prediction process calculates propensities of binding sites and then formulates a combination of sites scoring above a user-defined post-translational modification threshold.

SPECTRUM provides support for searching fixed, variable and blind post-translational modifications (PTMs) (Fig. 8). For *fixed* modifications, each instance of the implicated amino acid site was modified. For *variable* modifications^32^, the product of amino acid occurrence propensities within a certain enzyme binding site was obtained. An enzyme binding site could be a single or multi-residue substrate site containing the amino acid to be modified. Binding sites scoring above a user-specified threshold were selected for onward modifications (see equation (15)). In case multiple sites were shortlisted, all combinations of modified protein were created.

15 $$PTM\_Score > PTM\_Thr$$

where,

*PTM*_*Score* is the product of amino acid occurrence propensities within the binding site, and *PTM*_*Thr* is the user-specified threshold selected for modifications.

### Datasets used for validating SPECTRUM’s search pipeline

SPECTRUM validation was performed using datasets from two published top-down proteomics experiments including a HeLa^41^ and an *Escherichia coli*^25^ dataset. The HeLa dataset, which was used in case study 1, comprised of 10 MS spectra of Hela Histone H4 protein obtained using a Q-FTICR hybrid mass spectrometer. The spectra were calibrated externally using an electron-capture dissociation (ECD) bovine ubiquitin spectrum. Case study II employed *Escherichia coli* K-12 MG1655 dataset, which was acquired using an LTQ Orbitrap Velos mass spectrometer in an alternating fragmentation setting. The resulting data comprised of two sets of spectra, each containing 2027 scans from collision-induced dissociation (CID) and electron-transfer dissociation (ETD), respectively. SPECTRUM was employed to search the two datasets and the results were compared with those obtained from ProSightPC^42^ (a commercial version of ProSight PTM 2.0^22^), TopPIC^25^, pTop^24^ and MSPathFinder^26^.

### Validating SPECTRUM results

Target-decoy approach^48,49^ was employed to estimate the false discovery rate (FDR). The decoy database was generated by shuffling the protein sequences followed by the incorporation of three random amino acid mutations^26,50^. To further enhance the stability of FDR estimate, three decoy proteins were assembled for each protein entry in the target database. FDR was computed using equation^48^ (16). To estimate the statistical significance of each candidate protein, E-values were computed using an adaptation of generating function method^51^. For that, the probability of each amino acid is computed in the database. These amino acid probabilities are then used to calculate the probability of each protein sequence in the database. Using the number of spectral matches, the spectral probability^51^ of each sequence is then computed using equation (17). This is followed by an adjustment^51^ for truncation and computation of E-value using equation (18).

16 $$FDR=\,\frac{2\,\ast DB+DO}{TO+TB+DB}$$

17 $$SpectralProbability=\sum Probability\_of\_Sequences(spectralMatches\ge t)$$

18 $$EValue=0.693\ast SpectralProbability$$

### Data conversion to supported file formats

SPECTRUM requires experimental data in standardized input file formats. These formats include Mascot Generic Format (MGF)^7^, eXtensible Markup Language (XML) file containing mass to charge ratios (mz) and relative abundances (mzXML)^36,43^, and Mass Spectrometry Markup Language (mzML)^38,39^. Raw data files such as Thermo Xcalibur ‘.raw’, ABI/Sciex ‘.WIFF’ and Bruker ‘.YEP’, therefore, need to be converted into the aforementioned formats. For that, file format conversion and deconvolution tools such as MS-Convert^52^ and MS-Deconv^53^ can be employed. mzXML and mzML files with centroided and peak-picked data, obtained using MS-Convert^52^, can be imported into SPECTRUM. SPECTRUM then relies on MS-Deconv^53^ and OpenMS^54^ to extract monoisotopic peak lists. Deconvolved MGF files containing monoisotopic peaks are automatically converted into searchable flat text files, using a custom file reader that has been implemented in SPECTRUM.

## Supplementary information

Supplementary Information_Unmarked

Supplementary Data S1

Supplementary Data S2

Supplementary Data S3

Supplementary Data S4

Supplementary Data S5

Supplementary Table S1 - Search Parameters - SPECTRUM vs pTop

Supplementary Table S2 - Summary Results - SPECTRUM vs pTop

Supplementary Table S3 - Complete Results - SPECTRUM vs pTop

Supplementary Table S4 - Search Parameters - SPECTRUM vs TopPIC

Supplementary Table S5 - Summary Results - SPECTRUM vs TopPIC

Supplementary Table S6 - Complete Results - SPECTRUM vs TopPIC

Supplementary Table S7 - Search Parameters - SPECTRUM vs ProSightPC

Supplementary Table S8 - Summary Results - SPECTRUM vs ProSightPC

Supplementary Table S9 - Complete Results - SPECTRUM vs ProSightPC

Supplementary Table S10 - Summary Results - Overall (SPECTRUM vs ProSight PC, TopPIC, and pTop)

Supplementary Table S11 - Blind PTM - Spectrum vs TopPIC

Supplementary Table S12 - Parameter Sensitivity Analysis

Supplementary Table S13 - Search Parameters - with PST - SPECTRUM vs MSPathFinder vs pTop

Supplementary Table S14 - Search Parameters - without PST - SPECTRUM vs TopPIC vs MSPathFinder

Supplementary Table S15 - Summary Results - Overall

Supplementary Table S16 - Complete Results - SPECTRUM with PSTs - CID - Decoy Search

Supplementary Table S17 - Complete Results - SPECTRUM with PSTs - CID - Target Search

Supplementary Table S18 - Complete Results - SPECTRUM with PSTs - ETD - Decoy Search

Supplementary Table S19 - Complete Results - SPECTRUM with PSTs - ETD - Target Search

Supplementary Table S20 - Complete Results - SPECTRUM without PSTs - CID - Decoy Search

Supplementary Table S21 - Complete Results - SPECTRUM without PSTs - CID - Target Search

Supplementary Table S22 - Complete Results - SPECTRUM without PSTs - ETD - Decoy Search

Supplementary Table S23 - Complete Results - SPECTRUM without PSTs - ETD - Target Search

Supplementary Table S24 - Feature Comparison - SPECTRUM vs Other TDP Tools

Supplementary Table S25 - File Format Comparison - SPECTRUM vs Other TDP Tools

## References

[1] Wasinger VC (1995). Progress with gene‐product mapping of the Mollicutes: Mycoplasma genitalium. Electrophoresis.

[2] Han X, Aslanian A, Yates JR (2008). Mass spectrometry for proteomics. Curr. Opin. Chem. Biol..

[3] Smith LM (2013). Proteoform: a single term describing protein complexity. Nat. Methods.

[4] Zhang Y, Fonslow BR, Shan B, Baek M-C, Yates JR (2013). Protein analysis by shotgun/bottom-up proteomics. Chem. Rev..

[5] Gundry, R. L. *et al*. Preparation of Proteins and Peptides for Mass Spectrometry Analysis in a Bottom‐Up Proteomics Workflow. *Curr. Protoc. Mol. Biol*. 10.25. 1–10.25. 23 (2009). 10.1002/0471142727.mb1025s88 PMC2905857 19816929

[6] Qian W-J (2005). Probability-based evaluation of peptide and protein identifications from tandem mass spectrometry and SEQUEST analysis: the human proteome. J. Proteome Res..

[7] Perkins DN, Pappin DJC, Creasy DM, Cottrell JS (1999). Probability-based protein identification by searching sequence databases using mass spectrometry data. Electrophoresis.

[8] Gasteiger E (2003). ExPASy: the proteomics server for in-depth protein knowledge and analysis. Nucleic Acids Res..

[9] Gattiker A, Bienvenut WV, Bairoch A, Gasteiger E (2002). FindPept, a tool to identify unmatched masses in peptide mass fingerprinting protein identification. Proteomics.

[10] Gluck F (2013). EasyProt—an easy-to-use graphical platform for proteomics data analysis. J. Proteomics.

[11] Tran JC (2011). Mapping intact protein isoforms in discovery mode using top-down proteomics. Nature.

[12] LeDuc RD (2004). ProSight PTM: an integrated environment for protein identification and characterization by top-down mass spectrometry. Nucleic Acids Res..

[13] Wu, S. *et al*. Top-down characterization of the post-translationally modified intact periplasmic proteome from the bacterium Novosphingobium aromaticivorans. *Int. J. Proteomics* **2013** (2013). 10.1155/2013/279590 PMC3608174 23555055

[14] El-Aneed A, Cohen A, Banoub J (2009). Mass spectrometry, review of the basics: electrospray, MALDI, and commonly used mass analyzers. Appl. Spectrosc. Rev..

[15] Monge ME, Harris GA, Dwivedi P, Fernández FM (2013). Mass spectrometry: recent advances in direct open air surface sampling/ionization. Chem. Rev..

[16] Yates JR, Kelleher NL (2013). Top down proteomics. Anal Chem.

[17] Armirotti A, Damonte G (2010). Achievements and perspectives of top-down proteomics. Proteomics.

[18] Zhou M, Veenstra T (2008). Mass spectrometry: m/z 1983-2008. Biotechniques.

[19] Fornelli, L. *et al*. Top-down proteomics: Where we are, where we are going? *J. Proteomics* (2017).

[20] Cai W, Tucholski TM, Gregorich ZR, Ge Y (2016). Top-down proteomics: technology advancements and applications to heart diseases. Expert Rev. Proteomics.

[21] Gregorich ZR, Ge Y (2014). Top‐down proteomics in health and disease: Challenges and opportunities. Proteomics.

[22] Zamdborg L (2007). ProSight PTM 2.0: improved protein identification and characterization for top down mass spectrometry. Nucleic Acids Res..

[23] Liu X (2012). Protein identification using top-down spectra. Mol. Cell. Proteomics.

[24] Sun R-X (2016). pTop 1.0: a high-accuracy and high-efficiency search engine for intact protein identification. Anal. Chem..

[25] Kou Q, Xun L, Liu X (2016). TopPIC: a software tool for top-down mass spectrometry-based proteoform identification and characterization. Bioinformatics.

[26] Park J (2017). Informed-Proteomics: open-source software package for top-down proteomics. Nat. Methods.

[27] Pesavento JJ, Kim Y-B, Taylor GK, Kelleher NL (2004). Shotgun annotation of histone modifications: a new approach for streamlined characterization of proteins by top down mass spectrometry. J. Am. Chem. Soc..

[28] Tsur, D., Tanner, S., Zandi, E., Bafna, V. & Pevzner, P. A. Identification of post-translational modifications via blind search of mass-spectra. In *Computational Systems Bioinformatics Conference, 2005. Proceedings. 2005 IEEE* 157–166 (IEEE, 2005). 10.1109/csb.2005.34 16447973

[29] Tanner S (2005). InsPecT: identification of posttranslationally modified peptides from tandem mass spectra. Anal. Chem..

[30] Mann M, Wilm M (1994). Error-tolerant identification of peptides in sequence databases by peptide sequence tags. Anal. Chem..

[31] Eisenhaber, B. & Eisenhaber, F. Prediction of posttranslational modification of proteins from their amino acid sequence. *Data Min. Tech. Life Sci*. 365–384 (2010). 10.1007/978-1-60327-241-4_21 20221930

[32] Lu, C.-T. *et al*. DbPTM 3.0: an informative resource for investigating substrate site specificity and functional association of protein post-translational modifications. *Nucleic Acids Res*. gks1229 (2012). 10.1093/nar/gks1229 PMC3531199 23193290

[33] Eng JK, McCormack AL, Yates JR (1994). An approach to correlate tandem mass spectral data of peptides with amino acid sequences in a protein database. J. Am. Soc. Mass Spectrom..

[34] Cottrell JS, London U (1999). Probability-based protein identification by searching sequence databases using mass spectrometry data. Electrophoresis.

[35] Baumgardner LA, Shanmugam AK, Lam H, Eng JK, Martin DB (2011). Fast parallel tandem mass spectral library searching using GPU hardware acceleration. J. Proteome Res..

[36] Deutsch EW (2012). File formats commonly used in mass spectrometry proteomics. Mol. Cell. Proteomics.

[37] Pedrioli PGA (2004). A common open representation of mass spectrometry data and its application to proteomics research. Nat. Biotechnol..

[38] Turewicz, M. & Deutsch, E. W. In *Data mining in proteomics* 179–203 (Springer, 2011).

[39] Martens L (2011). mzML—a community standard for mass spectrometry data. Mol. Cell. Proteomics.

[40] MathWorks. MATLAB. Available at: https://www.mathworks.com (1994).

[41] Frank AM, Pesavento JJ, Mizzen CA, Kelleher NL, Pevzner PA (2008). Interpreting top-down mass spectra using spectral alignment. Anal. Chem..

[42] Inc., T. F. S. ProSightPC 4.0. Available at: http://proteinaceous.net/product/prosightpc-4-0/(2013).

[43] Lin SM, Zhu L, Winter AQ, Sasinowski M, Kibbe WA (2005). What is mzXML good for?. Expert Rev. Proteomics.

[44] Peng Y (2012). Top-down targeted proteomics for deep sequencing of tropomyosin isoforms. J. Proteome Res..

[45] Calligaris D, Villard C, Lafitte D (2011). Advances in top-down proteomics for disease biomarker discovery. J. Proteomics.

[46] Siuti N, Kelleher NL (2007). Decoding protein modifications using top-down mass spectrometry. Nat. Methods.

[47] Savaryn JP, Catherman AD, Thomas PM, Abecassis MM, Kelleher NL (2013). The emergence of top-down proteomics in clinical research. Genome Med..

[48] Aggarwal, S. & Yadav, A. K. In *Statistical Analysis in Proteomics* 119–128 (Springer, 2016).

[49] Navarro P, Vázquez J (2009). A refined method to calculate false discovery rates for peptide identification using decoy databases. J. Proteome Res..

[50] Park, J. K. *et al*. *Informed-Proteomics: Open Source Software Package for Top-Down Proteomics*. (Pacific Northwest National Laboratory (PNNL), Richland, WA (US), Environmental Molecular Sciences Laboratory (EMSL), 2017).

[51] Liu, X., Segar, M. W., Li, S. C. & Kim, S. Spectral probabilities of top-down tandem mass spectra. In *BMC genomics* **15**, S9 (BioMed Central, 2014). 10.1186/1471-2164-15-S1-S9 PMC4046700 24564718

[52] Chambers MC (2012). A cross-platform toolkit for mass spectrometry and proteomics. Nat. Biotechnol..

[53] Liu X (2010). Deconvolution and database search of complex tandem mass spectra of intact proteins a combinatorial approach. Mol. Cell. Proteomics.

[54] Röst HL (2016). OpenMS: a flexible open-source software platform for mass spectrometry data analysis. Nat. Methods.

