# Supplementary material for: SPECTRUM – A MATLAB Toolbox for Proteoform Identification from Top-Down Proteomics Data
Source: Sci Rep. 2019 Aug 2;9:11267. doi: 10.1038/s41598-019-47724-1 (PMC6677810; doi:10.1038/s41598-019-47724-1)
Supplement: Supplementary file 1 — Supplementary Information_Unmarked [file 41598_2019_47724_MOESM1_ESM.docx]

**Supplementary Information**

SPECTRUM – A MATLAB Toolbox for Proteoform Identification from Top-Down Proteomics Data

**Abdul Rehman Basharat^1^, Kanzal Iman^1^, Muhammad Farhan Khalid^1^, Zohra Anwar^1^, Rashid Hussain^1^, Humnah Gohar Kabir^1^, Maria Tahreem^1^, Anam Shahid^1^, Maheen Humayun^1^, Hira Azmat Hayat^2^, Muhammad Mustafa^1^, Muhammad Ali Shoaib^1^, Zakir Ullah^3^, Shamshad Zarina^4^, Sameer Ahmed^1^, Emad Uddin^5^, Sadia Hamera^6^, Fayyaz Ahmad^7^, Safee Ullah Chaudhary^1, *^**

^1^Biomedical Informatics Research Laboratory, Department of Biology, Lahore University of Management Sciences, Lahore, Pakistan

^2^Department of Computer Science, Lahore University of Management Sciences, Lahore, Pakistan
^3^King Abdullah University of Science and Technology, Thuwal, Saudi Arabia
^4^National Center for Proteomics, University of Karachi, Karachi, Pakistan

^5^Department of Mechanical Engineering, National University of Sciences and Technology, Islamabad, Pakistan

^6^Institute of Life Sciences, University of Rostock, Rostock, Germany

^7^Department of Statistics, University of Gujrat, Gujrat, Pakistan

**Table of Contents**

[A. Supplementary Methods 3](#_Toc535950086)

[1. Intact Protein Mass Tuner 3](#_Toc535950087)

[2. Computing Theoretical Mass of a Protein 4](#_Toc535950088)

[3. Extraction of Peptide Sequence Tags 5](#_Toc535950089)

[4. Spectral Generation and Comparison 6](#_Toc535950090)

[5. FDR Estimation Process 6](#_Toc535950091)

[B. Supplementary Results 7](#_Toc535950092)

[1. Case Study I – Evaluation of SPECTRUM Search with Known Target Protein 7](#_Toc535950093)

[2. Case Study II – Evaluation of SPECTRUM Search with Unknown Target Protein 8](#_Toc535950094)

[C. Graphical User Interfaces (GUIs) of SPECTRUM 10](#_Toc535950095)

[D. Step-by-Step Guidelines for using SPECTRUM 15](#_Toc535950096)

[E. Availability 18](#_Toc535950097)

[F. Video Tutorials 19](#_Toc535950098)

[G. Worked Examples 20](#_Toc535950099)

[1. Single File Search with Known Protein 20](#_Toc535950100)

[2. Batch Mode Search 24](#_Toc535950101)

[3. Uploading a New Database 28](#_Toc535950102)

[4. Search for Unknown PTMs using Blind-PTM search 30](#_Toc535950103)

[H. Feature Comparison 32](#_Toc535950104)

# **Supplementary Methods**

SPECTRUM is an open source and open architecture MATLAB toolbox for the analysis of top-down proteomics data. The proposed toolbox supports multiple data file formats and an intuitive a set of graphical user interfaces (GUIs) have been provided for protein search and visualization of results. The SPECTRUM pipeline includes initial filtering of protein database which is heavily dependent on an accurate whole protein mass (MS1). Towards increasing the accuracy of this step, SPECTRUM provides an intact protein mass tuner which employs MS2 data for tuning MS1. Onwards, the tuned mass is used for shortlisting candidate proteins from user-selected protein database. Next, *de novo* sequencing provides peptide sequence tags (PSTs) for further filtering of candidate proteins. Lastly, a spectral comparison is performed between the theoretical spectra of candidate proteins and MS2 data. Moreover, identification of post-translational modifications (PTMs) using statistically inferred binding sites, support for blind PTM search and the identification of truncated proteins has also been provided. The methodology employed in developing each salient component of the search pipeline is outlined below.

## Intact Protein Mass Tuner

The mathematical equations detailing on SPECTRUM’s intact protein mass tuner are given below (equations (1-7)).

$PK=\{MS_{1},MS_{2},INT\}$

Where,

$PK$is a set of MS data including mass to charge ratios (m/z) of intact proteins and its peptides along with their relative abundances, $MS_{1}$ is intact proteins mass, where $\left(\frac{m}{z}\right)_{p}$is the mass to charge ratio of *p*^th^ fragment in$MS_{2},$ $INT=\{\mathrm{int}_{1},\mathrm{int}_{2},...,\mathrm{int}_{p},...,\mathrm{int}_{n}\}$ where $\mathrm{int}{}_{p}$ is the intensity of *p*^th^ fragment in $INT$ corresponding to each $MS_{2}$, and $n=\text{ }size\text{ }(MS_{2})$.

Where,

$fps^{mz}$ is a set of m/z value summations for each fragment-pair.

Where,

$fps^{int}$ is a set of average relative abundances for each fragment-pair in $fps^{mz}$.

Where,

$FPS^{mz,int}$ is a set of elements in $fps^{mz}$ falling within the user defined mass tolerance and corresponding $fps^{int}$, and $Tol$ is user-defined intact protein mass tolerance.

$$Win_{p}=\{FPS^{mz,int}|seed_{p}-\frac{mass_{proton}}{2}\leq FPS^{mz,int}(fps^{mz})\leq seed_{p}+\frac{mass_{proton}}{2}\}$$

Where,

$Win_{p}$is a set of summations of m/z values and corresponding intensities falling within *p*^th^ window, $mass_{proton}$ is the mass of proton i.e. 1.007276 Da, and $seed_{p}$ is the seeding point of *p*^th^ sliding window.

$$Win_{selected}=\{(mz,intensity)\in Win_{p}|max(\left|mz\right|)\}$$

Where,

$Win_{selected}$ is a set of fragment-pair sums and corresponding intensities falling in the window with the maximum number of elements.

$$TunedMass=\frac{\sum_{i=1}^{k} (mz_{i}\times intensity_{i})}{\sum_{i=1}^{k} intensity_{i}}$$

Where,

$TunedMass$ is the tuned protein mass,$(mz,intensity)\in Win_{selected}$, and $k$ is total number of fragment-pairs in $Win_{selected}$.

## Computing Theoretical Mass of a Protein

The mathematical equation for calculating protein mass has been described below (equation (8)).

$$Mass_{theoretical}=\sum_{i=1}^{n} mass(ProteinSequence_{i})\text{ }+\text{ }<H_{2}O>$$

Where,

$Mass_{theoretical}$is theoretical protein mass calculated using protein sequence, $ProteinSequence$is a protein sequence containing standard amino acids, $mass(ProteinSequence_{i})$returns the monoisotopic molecular weight of *i* ^th^ amino acid, $n$ is number of amino acids in the protein sequence, and $<H_{2}0>$ is monoisotopic mass of water.

## Extraction of Peptide Sequence Tags

The process of extracting peptide sequence tags (PSTs) from MS2 data has been described below in equations (9-13).

Where,

$fpd^{mz}$ is a set of m/z value differences for each fragment-pair, and $\left(\frac{m}{z}\right)_{n}$ is the mass to charge ratio of *n*^th^ fragment in $MS_{2}$.

Where,

$fpd^{int}$ is a set of average relative intensities for each fragment-pair in $fpd^{mz}$, and $int{}_{n}$ is the relative abundance of *n*^th^ fragment in $INT$ corresponding to $MS_{2}$ values.

$$FPD^{mz,int}=\{(fpd^{mz},fpd^{int})|mass_{AA}-Tol\leq fpd^{mz}\leq mass_{AA}+Tol\}$$

Where,

$FPD^{mz,int}$ is a set of elements in $fpd^{mz}$matching standard amino acids masses within user defined tolerance and corresponding intensities from in$fpd^{int}$, $Tol$ is user defined peptide sequence tag hop tolerance, and $mass_{AA}$ is a set of monoisotopic masses of standard amino acids.

$$AA{}^{tag}=\{AminoAcid|mass(AminoAcid)\in FPD^{mz,int}(fpd^{mz})\}$$

Where,

$AA^{tag}$ is the set of amino acid tags obtained corresponding to m/z values in $FPD$.

Let, and be the start and end positions of hops $h1$ and $h2$ corresponding to the masses of amino acids $X_{1}$ and $X_{2}$ $,$ respectively.

Where,

$X$ is an amino acid tag, and $PST$ is the peptide sequence tag obtained after collation of amino acids having equal start and end peaks.

## Spectral Generation and Comparison

The process of generating theoretical spectra has been described below in Supplementary equations (14-15).

$$N\_term\_Ion_{i}=\sum_{i=1}^{n} mass(ProteinSequence_{i})$$

$$C\_term\_Ion_{i}=MolW-N\_term\_Ion_{i}+<NeutralLoss>$$

Where,

$mass(ProteinSequence_{i})$returns the mass of an amino acid present at i^th^ position in the protein,$<NeutralLoss>$ represents the monoisotopic mass of fragment lost as neutral molecule, and $n$ is number of amino acids in the protein sequence.

## FDR Estimation Process

For any given score threshold, a protein is classified as a target only (TO) or decoy only (DO). If target protein’s score is greater than the score threshold, the protein entry is considered TO otherwise it is classified as DO. In the case where both target and decoy hits score above the threshold, they are distinguished based on which of the two has a higher score. If target protein has better score it is called target better (TB) otherwise we classify it as decoy better (DB). Onwards, FDR computation is performed using equation (16), below.


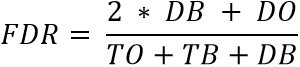
 (16)

# Supplementary Results

## Case Study I – Evaluation of SPECTRUM Search with Known Target Protein

A case study was performed to validate SPECTRUM by using a HeLa spectral dataset^1^ of Histone H4 protein. Alongside, we also undertook a blind PTM search and a parameter sensitivity analysis. SPECTRUM’s blind PTM search module was compared with TopPIC by allowing for mass shifts. SPECTRUM characterized seven PTMs from three data files while TopPIC only reported unknown mass shifts for seven identifications without translating them into PTMs. The detailed results are provided below in Supplementary Table S8.

**Supplementary Table S8. Comparison of blind PTM search results from SPECTRUM and TopPIC.** SPECTRUM reported PTMs from three out of ten data files while TopPIC reported unknown mass shifts for seven data files without translating them into PTMs.

To analyze the specific impact of intact mass, PST and *in silico* components on SPECTRUM’s pipeline, a parameter sensitivity analysis was conducted. The results obtained indicated that variations in PST length have a significant impact on protein identification as compared to that in mass tolerance, indicating a minimal effect. The results have been tabulated in Supplementary Table S9.

**Supplementary Table S9. Parameter Sensitivity Analysis.** Parameter sensitivity analysis was performed on SPECTRUM’s intact mass, PST and *in silico* comparison components. Intact protein mass and *in silico* spectral comparison tolerances was varied between 250-2000 Da and 15-25 ppm, respectively. PST lengths of 3-6 and 4-6 were considered.

## Case Study II – Evaluation of SPECTRUM Search with Unknown Target Protein

A case study was performed to validate SPECTRUM by searching a published *Escherichia coli* dataset^2^. The table below indexes the supplementary tables S16-S23 with summary results for each file along with complete search results (Supplementary Data S4), for the case study.

| **Dataset** | **PST Status** | **Database** | **Result** | **Supplementary Data File Name** |
| --- | --- | --- | --- | --- |
| **E-coli – CID Dataset** | **PST** | **Target** | Summary | Supplementary Table S17 - Complete Results - SPECTRUM with PSTs - CID - Target Search |
|  |  |  | Complete | Supplementary Data S4 - Case Study 2 - Complete Results (Complete Result Files - SPECTRUM with PSTs - CID - Target Search) |
|  |  | **Decoy** | Summary | Supplementary Table S16 - Complete Results - SPECTRUM with PSTs - CID - Decoy Search |
|  |  |  | Complete | Supplementary Data S4 - Case Study 2 - Complete Results (Complete Result Files - SPECTRUM with PSTs - CID - Decoy Search ) |
|  | **Without PST** | **Target** | Summary | Supplementary Table S21 - Complete Results - SPECTRUM without PSTs - CID - Target Search |
|  |  |  | Complete | Supplementary Data S4 - Case Study 2 - Complete Results (Complete Result Files - SPECTRUM without PSTs - CID - Target Search) |
|  |  | **Decoy** | Summary | Supplementary Table S20 - Complete Results - SPECTRUM without PSTs - CID - Decoy Search |
|  |  |  | Complete | Supplementary Data S4 - Case Study 2 - Complete Results (Complete Result Files - SPECTRUM without PSTs - CID - Decoy Search ) |
| **E-coli – ETD Dataset** | **PST** | **Target** | Summary | Supplementary Table S19 - Complete Results - SPECTRUM with PSTs - ETD - Target Search |
|  |  |  | Complete | Supplementary Data S4 - Case Study 2 - Complete Results (Complete Result Files - SPECTRUM with PSTs - ETD - Target Search) |
|  |  | **Decoy** | Summary | Supplementary Table S18 - Complete Results - SPECTRUM with PSTs - ETD - Decoy Search |
|  |  |  | Complete | Supplementary Data S4 - Case Study 2 - Complete Results (Complete Result Files - SPECTRUM with PSTs - ETD - Decoy Search) |
|  | **Without PST** | **Target** | Summary | Supplementary Table S23 - Complete Results - SPECTRUM without PSTs - ETD - Target Search |
|  |  |  | Complete | Supplementary Data S4 - Case Study 2 - Complete Results (Complete Result Files - SPECTRUM without PSTs - ETD - Target Search) |
|  |  | **Decoy** | Summary | Supplementary Table S22 - Complete Results - SPECTRUM without PSTs - ETD - Decoy Search |
|  |  |  | Complete | Supplementary Data S4 - Case Study 2 - Complete Results (Complete Result Files - SPECTRUM without PSTs - ETD - Decoy Search) |

# Graphical User Interfaces (GUIs) of SPECTRUM

| **SPECTRUM – Main Graphical User Interface (GUI)** |
| --- |
| SPECTRUM Main GUI provides entry-point to users for setting up spectral data, protein database and general search parameters. |
| **SPECTRUM – Intact Protein Mass Tuner** |
| GUI to tune intact protein mass towards augmenting MS1 measurements for scoring and filtering protein databases. |
| **SPECTRUM – GUI for Setting-up PST Search Parameters** |
| GUI to provide peptide sequence tag search parameters for scoring and filtering protein databases. |
| **SPECTRUM – GUI for Selecting Fragmentation Ions and Mass Mode** |
| GUI to specify special ‘Fragmentation Ions’ (optional) and ‘Mass Mode’ (protonated/Neutral) in the search process. |
| **SPECTRUM – GUI for Setting-up Chemical and Terminal Modifications** |
| GUI to specify ‘Chemical Modifications’ along with ‘Terminal modifications’. |
| **SPECTRUM – GUI for Setting-up Scoring Component Weights** |
| GUI to tailor final scoring scheme wherein users can tune weights for individual component scores to obtain the final score. |
| **SPECTRUM – Summary Result GUI** |
| A GUI for displaying a list of ranked proteins along with their corresponding intact masses, terminal modifications and final scores. |
| **SPECTRUM – Detailed Results GUI** |
| Using the ‘Detailed Protein View’ GUI, users can view detailed information of each protein hit such as predicted modifications, reported peptide sequence tags, spectral visualization and matched theoretical fragments. |

# Step-by-Step Guidelines for using SPECTRUM

|  | | | | | | | | |
| --- | --- | --- | --- | --- | --- | --- | --- | --- |
| **Step 1:** Run the main GUI of the toolbox and input the ‘Project Title’. | |  | |  | | | **Step 2:** Choose the processing mode as either ‘Single Search’ or ‘Batch Mode’. For the batch run choose the ‘File Type’. | |
|  | | | | | | | | |
| **Step 3:** Choose the directory for the Database, peak list file and output folder. | |  | |  | | | **Step 4:** Select whether to ‘Filter Protein Database’ for intact protein mass and tune the whole protein molecular weight. |
|  | | | | | | | | |
| **Step 5:** Select whether to allow for ‘Truncated Proteoforms’. | |  | |  | | | **Step 6:** Select the ‘Fragmentation’ type. | |
|  | | | | | | | | |
| **Step 7:** Select ‘Experimental Detail’ to include special ions in protein search. Select ‘Mass Mode’ of experimental data. |  | |  | | **Step 8:** Select the desired ‘Fixed’ and ‘Variable’ post translational modifications or search for ‘Blind PTMs’. | | | |
|  | | | | | | | | |
| **Step 9:** Select cystine and methionine ‘Chemical Modifications’ along with desired ‘Terminal Modifications’. |  | |  | | **Step 10:** Set the fragments and molecular weight ‘Tolerance’ with desired tolerance measurement. | | | |
|  | | | | | | | | |
| **Step 11:** If user selects molecular weight tuning, the molecular weight estimation GUI will open. Using it, the intact mass can be estimated within tolerance. | |  | |  | | **Step 12:** In the PST GUI, add the length of sequence tag, ‘Tolerance For Each Hop’ and ‘Tolerance For Whole PST’ to check for and score PST’s in the peak list file. | | | |
|  | | | | | | | | |
| **Step 13:** Select the weight of each component towards final scoring |  | |  | | **Step 14:** This GUI will show summary result. To see detailed results for a protein, select ‘Detailed View’. | | | |
|  | | | | | | | | |
| **Step 15:** Detailed View of the protein selected in Step 14. |  | |  | | **Step 16:** This GUI is for spectral visualization of theoretical and experimental matches and mass differences. | | | |

|  | **Step 17:** This GUI will show number of matched fragments. |  |
| --- | --- | --- |

# Availability

| **Item** | **Description** | **Link** |
| --- | --- | --- |
| **Project Home** | URL link for SPECTRUM project home | <https://github.com/BIRL/SPECTRUM> |
| **Downloads** | Downloadable version of SPECTRUM | <https://github.com/BIRL/SPECTRUM/archive/master.zip> |
| **User’s Manual** | SPECTRUM User manual can be found here. | <https://github.com/BIRL/SPECTRUM/blob/master/Documentation/User%20Manual.pdf> |
| **Pull Requests** | Pull Requests page is available at this link where codes for SPECTRUM are collaborated. | <https://github.com/BIRL/SPECTRUM/pulls> |
| **Issues** | Issues and related discussions can be made at the issues page for SPECTRUM. | <https://github.com/BIRL/SPECTRUM/issues> |
| **Dataset 1 – HeLa** | Sample Data files used for performing SPECTRUM case study 1 are available at the following link. | <https://github.com/BIRL/SPECTRUM/tree/master/SampleDataandResults/CaseStudy1> |
| **Dataset 2 –**  ***E. Coli*** | Sample Data files used for performing SPECTRUM case study 2 are available at the following link. | <https://github.com/BIRL/SPECTRUM/tree/master/SampleDataandResults/CaseStudy2> |
| **Example Files** | A sample raw data file as well as its mzXML, mzML, MGF and Flat Text versions for searching with SPECTRUM is available at the following link. | [https://github.com/BIRL/SPECTRUM/tree/master/SampleDataandResults/ExampleFile/](https://github.com/BIRL/SPECTRUM/tree/master/SampleDataandResults/ExampleFile)  Note: SPECTRUM does not support space character in directory or file names. |
| **SPECTRUM Executable** | Public Mega folder containing the SPECTRUM Executable file (SPECTRUM.exe) for 64-bit Windows® operating system | [https://mega.nz/#F!x6BUzTTK!6Z-hSUbAQV_e8_VGXzSWbw](https://mega.nz/%23F!x6BUzTTK!6Z-hSUbAQV_e8_VGXzSWbw) |

# Video Tutorials

| **Tutorial Description** | **Playlist Title / Video Title in Playlist** |
| --- | --- |
| **Complete Playlist** - The following instructional videos have been made available in the form of a playlist titled “SPECTRUM” at the link: <https://www.youtube.com/playlist?list=PLaNVq-kFOn0YH6DpEMlXuxGwz8y7abZfQ> | SPECTRUM Version 1.0.0.0: A MATLAB Toolbox for Protein Identification from Top-Down Proteomics Data |
| **Download and Launch SPECTRUM** | Video Tutorial 01 – Downloading, Installing and Running SPECTRUM |
| **Raw to mzXML File Format Conversion** | Video Tutorial 02 - Raw to mzXML file format conversion |
| **Raw to mzML File Format Conversion** | Video Tutorial 03 - Raw to mzML file format conversion |
| **MzXML to MGF File Format Conversion** | Video Tutorial 04 - MzXML to MGF file format conversion |
| **MGF to Flat Text File Format Conversion** | Video Tutorial 05 - MGF to Flat Text file format conversion |
| **Single Mode Search** | Video Tutorial 06 - Single Mode Search |
| **Batch Mode Search** | Video Tutorial 07 - Batch Mode Search (Flat Text Files) |
| **Downloading and Using Protein Databases** | Video Tutorial 08 - Downloading and Using Databases |
| **Performing Search for mzXML File** | Video Tutorial 09 - Performing Search for mzXML File |
| **Performing Search for mzML File** | Video Tutorial 10 - Performing Search for mzML File |
| **Performing Search for MGF File** | Video Tutorial 11 - Performing Search for MGF File |
| **Computing FDR and Interpreting Results** | Video Tutorial 12 - Interpreting Results for Case Study 2 |

# Worked Examples

To exemplify the use of SPECTRUM, several worked out examples have been assembled and provided below.

## Single File Search with Known Protein

**Step 1 –** Enter the Project title and select ‘Single Search’ button. Browse for Data file and select MS data file (*.txt) for analysis. Browse for the folder containing database(s). Select Database File (e.g. *uniprot.fasta*).

**Step 2** – Click ‘Filter Protein Database’ to filter protein database on the basis of MS1 and ‘Truncated Proteoforms’ to allow search for truncated proteoforms.

**Step 3** – Select fragmentation type and click ‘Experimental Detail’ to provide experimental search details (Mass Mode and Type of Special Ions).

**Step 4** – Browse to select for the folder for saving ‘Search Parameters’. Browse to select for the folder to save ‘Results’. Select ‘Auto Tune’ to allow for tuning of MS1 using MS2 data. Enter protein mass, peptide and PTMs tolerances.

**Step 5** – Click ‘Other Modifications’ to select for ‘Chemical and Terminal modifications’.

**Step 6** – Select ‘Fixed’ and ‘Variable’ PTMs or click ‘Blind PTM Search’ to allow search for unknown post-translational modifications. Click ‘Next’ to proceed.

**Step 7** – Select ‘Filter Protein Database using PSTs’ to allow for peptide sequence tag-based search. Select for PST length and tolerance. Click ‘Next’ to proceed. Select ‘Scoring Components Weightage’ and click ‘Next’.

**Step 8** – Search Results will be displayed. Click ‘view’ for detailed results.

## Batch Mode Search

**Step 1 –** Enter the Project title and select ‘Batch Mode’ button. Browse for the folders containing data files for analysis and select the ‘File type’. Browse for the folder containing database(s). Select Database File (e.g. *uniprot.fasta*).

**Step 2** – Click ‘Filter Protein Database’ to filter protein database on the basis of MS1 and ‘Truncated Proteoforms’ to allow search for truncated proteoforms.

**Step 3** – Select fragmentation type and click ‘Experimental Detail’ to provide experimental search details (Mass Mode and Type of Special Ions).

**Step 4** – Browse to select for the folder for saving ‘Search Parameters’. Browse to select for the folder to save ‘Results’. Select ‘Auto Tune’ to allow for tuning of MS1 using MS2 data. Enter protein mass, peptide and PTMs tolerances.

**Step 5** – Click ‘Other Modifications’ to select for ‘Chemical and Terminal modifications’.

**Step 6** – Select ‘Fixed’ and ‘Variable’ PTMs or click ‘Blind PTM Search’ to allow search for unknown post-translational modifications. Click ‘Next’ to proceed.

**Step 7** – Select ‘Filter Protein Database using PSTs’ to allow for peptide sequence tag-based search. Select for PST length and tolerance. Click ‘Next’ to proceed. Select ‘Scoring Components Weightage’ and click ‘Next’.


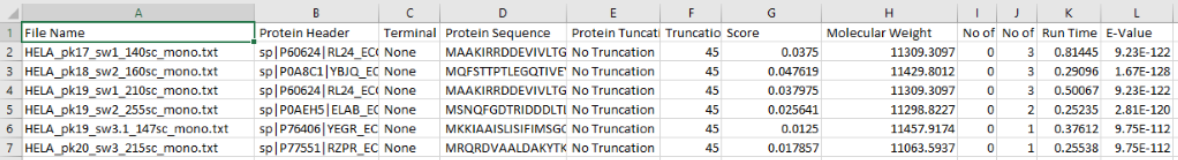
**Step 8** – Search Results will be saved as an excel sheet. Open file to view results.

## Uploading a New Database

**Step 1** – Go to UniProt ^3^ (<http://www.uniprot.org/>) to download a new database. Select for the organism (e.g. Rat) from the left to download its database.

**Step 2** – Select ‘Download all’ in ‘FASTA’ format and click ‘Go’ to download a ‘compressed’ file.

**Step 3** – Extract file and move the (*.fasta) file to ‘databases’ folder within SPECTRUM Toolbox folder.

**Step 4** – Browse for the ‘databases’ folder containing the downloaded database. Select downloaded Database File (e.g. *uniprot-rat.fasta*).

## Search for Unknown PTMs using Blind-PTM search

**Step 1** – Follow the steps mentioned in ‘Single Mode’ or ‘Batch mode’ search as required. Click ‘Blind PTM search’ to search for unknown modifications.

**Step 2** – Search results will be displayed. Click ‘view’ for detailed results. Location of unknown modifications along with their type will be present at the top of respective amino acid.

**Step 3** – Click ‘Legends’ to view details of modifications (A). Click ‘Experimental and Theoretical Fragment Matches’ to view details of fragment matches (B). Click ‘Mass Spectra’ for visual inspection of mass spectra (C). Click ‘Amino acid Abbreviations Chart’ to view amino acids and their three-letter and one-letter abbreviation (D).

# Feature Comparison

A feature comparison between SPECTRUM, MASH Suite Pro^4^, MSPathFinder^5^, ProSightPC^6,7^. TopPIC^2^ and pTop^8^ is provided in the table below.

**Supplementary Table S24. Comparison of features offered by SPECTRUM and contemporary tools.**

A table summarizing the comparison of file formats supported by SPECTRUM as well as other top-down proteomics tools including MSPathFinder^5^, ProsightPC^6^, TopPIC^2^ and pTop^8^ is as follows.

| **Tools** | **Formats Supported** | | | | | | | **Peak Used** |
| --- | --- | --- | --- | --- | --- | --- | --- | --- |
|  | **Raw** | **msalign** | **Text** | **MGF** | **mzXML** | **mzML** | **PUF** |  |
| *MSPathFinder* |  |  |  |  |  |  |  | Monoisotopic |
| *Prosight PC* |  |  |  |  |  |  |  | Monoisotopic |
| *TopPIC* |  |  |  |  |  |  |  | Monoisotopic |
| *pTop* |  |  |  |  |  |  |  | Monoisotopic |
| ***SPECTRUM*** |  |  |  |  |  |  |  | Monoisotopic |
|  |  |  |  |  |  |  |  |  |
| ** PUF - ProsightPC Upload Format* | | | |  |  |  |  |  |

**Supplementary Table S25. Comparison of input file formats offered by SPECTRUM and contemporary tools.**

**Supplementary References**

1. Frank, A. M., Pesavento, J. J., Mizzen, C. A., Kelleher, N. L. & Pevzner, P. A. Interpreting top-down mass spectra using spectral alignment. *Anal. Chem.* **80,** 2499–2505 (2008).

2. Kou, Q., Xun, L. & Liu, X. TopPIC: a software tool for top-down mass spectrometry-based proteoform identification and characterization. *Bioinformatics* **32,** 3495–3497 (2016).

3. Consortium, U. UniProt: the universal protein knowledgebase. *Nucleic Acids Res.* **45,** D158–D169 (2016).

4. Cai, W. *et al.* MASH Suite Pro: A comprehensive software tool for top-down proteomics. *Mol. Cell. Proteomics* **15,** 703–714 (2016).

5. Park, J. *et al.* Informed-Proteomics: open-source software package for top-down proteomics. *Nat. Methods* **14,** 909 (2017).

6. Inc., T. F. S. ProSightPC 4.0. (2013). Available at: http://proteinaceous.net/product/prosightpc-4-0/.

7. Zamdborg, L. *et al.* ProSight PTM 2.0: improved protein identification and characterization for top down mass spectrometry. *Nucleic Acids Res.* **35,** W701–W706 (2007).

8. Sun, R.-X. *et al.* pTop 1.0: a high-accuracy and high-efficiency search engine for intact protein identification. *Anal. Chem.* **88,** 3082–3090 (2016).
